# Unveiling long-term prenatal nutrition biomarkers in beef cattle via multi-tissue and multi-OMICs analysis

**DOI:** 10.1007/s11306-025-02384-3

**Published:** 2025-12-01

**Authors:** Guilherme Henrique Gebim Polizel, Ángela Cánovas, Wellison J. S. Diniz, German D. Ramírez-Zamudio, Aline Silva Mello Cesar, Heidge Fukumasu, Arícia Christofaro Fernandes, Édison Furlan, Miguel Henrique de Almeida Santana

**Affiliations:** 1 Department of Animal Science, GOPec, Faculty of Animal Science and Food Engineering, University of São Paulo, Av. Duque de Caxias Norte, 225, Pirassununga, SP 13635-900 Brazil; 2 Department of Animal Biosciences, Centre for Genetic Improvement of Livestock, University of Guelph, 50 Stone Road East, Guelph, ON Canada; 3 Department of Animal Sciences, College of Agriculture, Auburn University, Auburn, AL 36849 USA; 4 Department of Food Science and Technology, Luiz de Queiroz College of Agriculture, University of São Paulo, Av. Pádua Dias 11, Piracicaba, SP 13418-900 Brazil; 5 Department of Veterinary Medicine, Faculty of Animal Science and Food Engineering, University of São Paulo, Av. Duque de Caxias Norte, 225, Pirassununga, SP 13635-900 Brazil

**Keywords:** Beef prenatal nutrition, Metagenomics, Metabolomics, Systems biology, Transcriptomics

## Abstract

**Introduction:**

Maternal nutrition during gestation plays a crucial role in shaping offspring development, metabolism, and long-term health, yet the underlying molecular mechanisms remain poorly understood.

**Objectives:**

This study investigated potential biomarkers through multi-OMICs and multi-tissue analyses in offspring of beef cows subjected to different gestational nutrition regimes.

**Methods:**

A total of 126 cows were allocated to three groups: NP (control, mineral supplementation only), PP (protein-energy supplementation in the last trimester), and FP (protein-energy supplementation throughout gestation). Post-finishing phase, samples (blood, feces, ruminal fluid, fat, liver, and *longissimus* muscle/meat) were collected from 63 male offspring. RNA sequencing was performed on muscle and liver, metabolomics on plasma, fat, liver, and meat, and 16S rRNA sequencing on feces and ruminal fluid. Data were analyzed via DIABLO (mixOmics, R).

**Results:**

The muscle transcriptome showed strong cross-block correlations (|r| > 0.7), highlighting its sensitivity to maternal nutrition. Plasma glycerophospholipids (PC ae C30:0, PC ae C38:1, lysoPC a C28:0) were key biomarkers, particularly for FP. The PP group exhibited liver-associated markers (IL4I1 gene, butyrylcarnitine), reflecting late-gestation effects, while NP had reduced ruminal *Clostridia* (ASV151, ASV241), suggesting impaired microbial energy metabolism.

**Conclusions:**

This integrative multi-OMICs approach provided deeper insights than single-layer analyses, distinguishing nutritional groups and revealing tissue- and OMIC-specific patterns. These findings demonstrate the value of combining transcriptomic, metabolomic, and microbiome data to identify biomarkers linked to maternal nutrition in beef cattle.

**Supplementary Information:**

The online version contains supplementary material available at 10.1007/s11306-025-02384-3.

## Introduction

In tropical regions like Brazil, beef cattle are predominantly raised on pastures, and nutrition is a key factor influencing their performance. Cattle primarily graze on low-nutritive pastures that vary seasonally in quantity and quality, with sparse winter rainfall further reducing forage availability and nutritional value (Boval et al., 2015). A lack of adequate feed supplementation during this time can have a substantial negative effect on the animals’ performance (Santana et al., 2025). Throughout the production system, the first two-thirds of pregnancy are vital for key processes such as organogenesis (Funston et al., 2010) and myogenesis (Du et al., 2010), both of which are closely linked to important production and metabolic traits in beef cattle. The third trimester of gestation in beef cows, often coinciding with the dry winter season, is a critical phase for fetal development (da Silva et al., 2017). During this period, over 75% of fetal weight is gained (Bauman and Bruce Currie 1980), making proper feed supplementation essential for ensuring healthy calf growth.

Our team has conducted extensive research on the impact of maternal nutrition on various tissues and organs in beef cattle, including plasma (Junior et al. 2022; Polizel, Diniz, et al. 2025; Polizel et al. 2023), muscle (Cracco et al. 2024; Polizel, Cesar, et al. 2022; Polizel, Strefezzi, Polizel et al. 2021a, b), liver (Polizel, Cançado, Polizel et al. 2022a, b, 2024; Polizel, Santos, Polizel et al. 2025a, b), meat and subcutaneous fat (Fernandes, Beline, et al. 2023; Fernandes, Polizel, Fernandes et al. 2023a, b), and gastrointestinal tract (Dias et al. 2024; Polizel, Diniz, et al. 2025). Although we have observed long-term tissue-specific metabolic effects on offspring, a comprehensive multi-OMICs and multi-tissue approach can offer a deeper and more accurate understanding of the holistic impact of maternal nutrition on offspring metabolic programming. Molecular interactions in a biological system are fundamental to the complete understanding of cellular functions and the molecular basis of complex processes (Ardini et al. 2022; Cánovas et al. 2014).

With the decreasing costs and shorter timelines for large-scale -OMICs data generation, the accessibility of these technologies has greatly improved (Pinu et al., 2019). However, the high dimensionality of multi-OMICs datasets presents significant computational challenges, particularly when integrating data from diverse sources such as next-generation sequencing platforms (e.g., Illumina, PacBio), mass spectrometers (e.g., LC-MS, GC-MS), and genotyping technologies (e.g., SNP arrays, whole-genome sequencing). Moreover, the challenges for integration are also associated with the variations in measurement units (e.g., counts, abundance, peak intensity, SNPs) and data categories (e.g., qualitative, quantitative discrete, and quantitative continuous). These complexities require the development of advanced bioinformatics tools capable of handling, analyzing and integrating multi-OMICs data generated at transcriptome, metabolome and metagenome level across these complicating factors. While bioinformatics tools using dimensional reduction strategies can help mitigate this burden, their application remains limited, and there is a critical need for further tool development (Misra et al., 2019).

One widely used method for meta-dimensional multi-OMICs integration is the data integration analysis for biomarker discovery using latent components (DIABLO) from mixOmics R-Package (Singh et al., 2019). This approach is based on Projection to Latent Structures (PLS) and simultaneously identifies potential-OMICs biomarkers (e.g. genes, metabolites, microorganisms) while discriminating between treatment groups during the integration process.

This study represents the culmination of several years of research, utilizing comprehensive-OMICs data (including transcriptomics, metabolomics and metagenomics) and various tissue types and biological samples (i.e., liver, muscle and meat, subcutaneous fat, blood, feces, and ruminal fluid) to achieve a holistic understanding of the long-term effects of maternal nutrition on offspring. We hypothesize that maternal nutrition has long-term multi-OMICs and multi-tissue effects on beef cattle offspring. The objective was to identify potential biomarkers through a supervised machine learning technique (DIABLO) in the offspring associated with the maternal nutrition plane across three -OMICs (transcriptomics, metabolomics, and metagenomics) and seven tissue types and biological samples (plasma, muscle, liver, meat, subcutaneous fat, rumen fluid, and feces).

## Materials and methods

### Experimental design

The animals utilized in this study were sourced from the Faculty of Animal Science and Food Engineering (FZEA-USP) campus. All procedures involving the animals received approval from the FZEA-USP Institutional Animal Care and Use Committee (Protocol #1843241117). Pregnancy was confirmed thirty days following the artificial insemination of the cows, which were randomly assigned to receive semen from four different sires. The dams were categorized into three groups of 42 animals each, based on their age, body weight (BW), and body condition score, to ensure a well-balanced experimental design. These groups were maintained on grazing paddocks of *Urochloa brizantha* cv. Marandu, which were equipped with troughs for water and feed supplementation. The three groups of cows underwent different prenatal treatments: Not Programmed (NP, serving as the control), Partial Programming (PP), and Full Programming (FP). The NP group received only mineral supplements throughout their pregnancy, at a rate of 0.3 g/kg of their BW per day. In contrast, the PP group was given protein-energy supplementation during the final trimester of pregnancy, amounting to 3.0 g/kg of their BW per day. The FP group received the same protein-energy supplementation for an extended duration, commencing from the confirmation of pregnancy until calving.

The groups receiving protein-energy supplementation (PP and FP) were also provided with mineral supplementation, amounting to 0.3 g/kg of their BW per day. The formulation of the protein-energy supplement included the mineral components, as indicated in Table 1. Comprehensive details regarding the pasture conditions, as well as the phenotypic and metabolic impacts of the treatments (NP, PP, and FP) on the dams, have been previously described by Schalch Junior et al. (2022).

**Table 1:** Composition and nutritional content of the maternal supplement diet

Ingredients	Mineral supplement	Protein-energy supplement
Corn (%) Soybean meal (%) Dicalcium phosphate (%) Urea 45% (%) Salt (%) Minerthal 160 MD (%)* Total digestible nutrients (%) Crude protein (%) Non-protein nitrogen (%) Acid detergent fiber (%) Neutral detergent fiber (%) Fat (%) Calcium (g/kg) Phosphorus (g/kg)	35.00 - 10.00 - 30.00 25.00 26.76 2.79 - 1.25 4.29 1.26 74.11 59.38	60.00 30.00 - 2.50 5.00 2.50 67.55 24.78 7.03 4.76 11.24 2.61 6.20 7.24

*Mineral premix composition (Minerthal company): Calcium = 8.6 g/kg; Cobalt = 6.4 mg/kg; Copper = 108 mg/kg; Sulfur = 2.4 g/kg; Fluorine = 64 mg/kg; Phosphorus = 6.4 g/kg; Iodine = 5.4 mg/kg; Manganese = 108 mg/kg; Selenium = 3.2 mg/kg; Zinc = 324 mg/kg; Sodium monensin = 160 mg/kg (Polizel, Fantinato-Neto, et al. 2021)

Following calving, the provision of protein-energy supplements was stopped. All calves, irrespective of their prenatal nutritional treatment, were managed under identical health protocols and dietary plans until weaning, which occurred at approximately 240 ± 28 days. Throughout this interval, the cows received the same mineral supplementation (0.3 g/kg of BW) as they did during the gestation period and were maintained on an extensive grazing system consisting of *Urochloa brizantha* cv. Marandu pastures.

### Rearing and finishing phase

Post-weaning, animals were separated by sex and reared under the same nutritional management until 570 ± 28 days. During this period, young bulls grazed on *Urochloa brizantha* cv. Marandu pastures with free access to water and received energy supplements during the dry season and protein supplements during the wet season, adjusted to their body weight. At 570 ± 28 days, the finishing phase began, during which the bulls were fed three sequential diets: an adaptation diet for 15 days, followed by two finishing diets over 35 and 56 days, respectively. The finishing phase ended with slaughter at 676 ± 28 days, with average slaughter weights ranging from 591.2 kg to 602.6 kg. Slaughter and carcass processing were conducted in accordance with Brazilian Ministry of Agriculture, Livestock, and Supply (MAPA) guidelines. More detailed information about the rearing and finishing phase are available elsewhere (Dias et al. 2024; Fernandes, Beline, et al. 2023; Polizel, Strefezzi, et al. 2021).

### Tissue sample collection

Tissue samples were collected from 63 Nellore bulls. Plasma, liver, *longissimus thoracis* muscle, and rumen fluid were collected at the slaughterhouse within 15 min of slaughter (animals aged 676 ± 28 days). Fecal samples were collected 16 days prior to slaughter (660 ± 28 days of age). Subcutaneous fat and meat samples were collected 24 h post-slaughter, after *rigor mortis* was complete. For this study, five animals per treatment were randomly selected from the original 63, maintaining the same 15 animals across all biological samples and tissue types and -OMICs analyses.

Blood samples were aseptically collected from the jugular vein of each animal into EDTA-coated tubes to prevent coagulation. The samples were kept on ice and processed within one hour. Plasma was separated by centrifuging the blood at 3,000×g for 10 min at 4 °C. The plasma was carefully aspirated to avoid contamination and stored in sterile tubes at −80 °C to preserve its biochemical properties for future analysis. Liver tissue samples were collected from the distal portion of the left lobe, while *longissimus thoracis* (muscle and meat) and subcutaneous fat were sampled from the area between the 12th and 13th ribs. These tissues were immediately preserved in liquid nitrogen and later stored in an ultrafreezer (−80 °C). Rumen fluid was obtained post-evisceration, filtered through four layers of sterilized cheesecloth, and transferred to 20 mL conical tubes per animal. Fresh fecal samples were retrieved directly from the rectum using disposable gloves to minimize cross-contamination. Approximately 10–20 g of feces were placed in sterile, pre-labeled microtubes. Both fecal and rumen fluid samples were initially stored on ice packs and subsequently transferred to an ultrafreezer.

### Transcriptomics analyses and data pre-processing

RNA extraction was conducted using the TRIzol reagent following the manufacturer’s guidelines. Liver and muscle tissue samples weighing 100 mg were utilized for the isolation of RNA. The RNA concentration was assessed using a DS-11 spectrophotometer. The integrity of the RNA was analyzed with the Bioanalyzer 2100 (Agilent, Santa Clara, CA, USA), showing an average RNA integrity number (RIN) of 7.1 for liver and 7.2 for muscle, indicating a good quality. For the library preparation, RNA quantities ranging from 0.1 to 1 µg were used following the protocols outlined in the TruSeq Stranded mRNA Reference Guide. Library quantification was executed through quantitative PCR with the KAPA Library Quantification kit, and the average size of the library was evaluated using the Bioanalyzer 2100. Sequencing was performed on a single lane of a flow cell utilizing the TruSeq PE Cluster kit v3-cBot-HS and a paired-end sequencing strategy. The libraries were sequenced on the Illumina HiSeq 2500 instrument, and the TruSeq Stranded mRNA kit was applied as per the manufacturer’s instructions.

To assess the sequencing quality, the FastQC v.0.11.9 software was used. Subsequently, Seqyclean v.1.9.10 software was utilized to remove adapters that were introduced during the library preparation process (Zhbannikov et al. 2017). The alignment of the samples to the *Bos taurus* reference genome (ARS-UCD1.3.113) was conducted using STAR v.020201 software (Dobin et al. 2013) with default settings, incorporating an annotation file (ARS-UCD1.3.113), which produced a file detailing the number of reads associated with each gene (counts). EdgeR (Robinson et al. 2010) was used for filtering and normalization (log_2_CPM) of each dataset separately. Before the inclusion of genes in mixOmics, a final filtering step was conducted, retaining genes with a |logfoldchange| greater than 1.25 for at least one contrast, due to mixOmics’ limitations on the number of variables per block. This threshold was not applied as a statistical significance criterion but as a practical step to maintain model stability, given the relatively small number of differentially expressed genes in these datasets (Cracco et al. 2024; Polizel, Santos, et al. 2025). Ultimately, the final dataset contained 401 genes for the liver (Additional file 1) and 274 genes for the muscle (Additional file 2).

### Metagenomics analyses and data pre-processing

DNA was extracted using the Macherey Nagel NucleoSpin Tissue^®^ kit, and 16S rRNA gene sequencing was conducted on the MiSeq platform (Illumina, USA) using primers targeting the V3 to V4 region. The process involved two PCR steps: the first step amplified the 16S rRNA gene region with Illumina adapter sequences, and the PCR products were purified using AMPure XP beads and checked for size via agarose gel electrophoresis. In the second step, barcodes from the Nextera XT kit were added, followed by additional purification and validation of the library. The libraries were then quantified to ensure consistent pooling. The forward [S-D-Bact-0341-b-S-17 (5′-CCTACGGGNGGCWGCAG-3′)] and reverse primers [S-D-Bact-0785-a-A-21 (5′-GACTACHVGGGTATCTAATCC-3′)] (Klindworth et al., 2013) were used in combination with the Illumina adapter sequences to amplify the target region. The phi-X phage was added as a control, and the libraries were denatured to facilitate the sequencing.

The analysis of amplicon metagenomic data was conducted using the statistical software R v.4.4.0. The DADA2 v.1.32.0 pipeline was used to derive Amplicon Sequence Variants (ASVs) and to perform taxonomic classifications. Initially, we filtered and trimmed the raw sequencing reads to eliminate low-quality bases (quality score ≥ 30) and any adapter contamination. Subsequently, identical reads were consolidated through dereplication. The reads underwent denoising, merging, and filtering to eliminate artifacts associated with PCR and PhiX-related chimeras. The ASVs were quantified and taxonomically classified using the SILVA database of non-redundant sequences (v.138.2, nr99) (Pruesse et al., 2007). The data were organized into objects that included ASV quantifications, taxonomic annotations, samples and treatments (NP, PP, and FP) utilizing the phyloseq package v.1.48.0 (McMurdie & Holmes, 2013). The phyloseq package facilitated the removal of phyla represented by only a single feature and applied a prevalence filter (≥ 20%). Following all filtering procedures, the final datasets were log_2_(x + 1) transformed for integration into mixOmics. The fecal dataset (Additional file 3) and the ruminal fluid dataset (Additional file 4) yielded 258 and 302 microbial taxa at the genus level, respectively. The taxonomic tables for the fecal and ruminal fluid metagenomes are reported in Additional files 5 and 6, respectively.

### Metabolomics analyses and data pre-processing

Metabolites from meat, subcutaneous fat, and liver (solid tissues) were extracted using a cold solvent mixture consisting of 85% HPLC-grade ethanol and 15% phosphate buffer (0.01 M, pH 7.5), maintained at temperatures below 0 °C with dry ice. The samples were weighed, homogenized three times in the extraction solvent using a bead-based homogenizer (20 s at 5500 RPM), and then centrifuged (10,000 x g for 5 min). The supernatant was carefully collected and immediately stored in an ultra-low freezer (−80 °C) until metabolite analysis using the AbsoluteIDQ p180 Kit. For further details on the extraction protocol, refer to (Zukunft et al., 2018). No pre-processing was required for plasma samples.

The AbsoluteIDQ^®^ p180 Kit (Biocrates Life Sciences, Austria) was used to perform targeted metabolomics analysis on plasma samples, measuring 188 metabolites across various categories, including amino acids, biogenic amines, acylcarnitines, lysophosphatidylcholines, phosphatidylcholines, sphingolipids, and a monosaccharide. The analysis was conducted by Apex Science Company (Brazil) using the SCIEX 4000 series^®^ system. Amino acids and biogenic amines were quantified using HPLC-MS/MS with electrospray ionization, while other metabolites were analyzed by FIA-MS/MS. Metabolite identification and quantification were based on the MetIDQ™ software and its proprietary library, which uses validated reference standards and optimized methods for the p180 Kit. The data processing utilized isotopically labeled internal standards for accuracy and reproducibility. Biocrates ensures high-quality results with stringent quality control protocols. These include pre-determined limits of detection (LOD) for each metabolite, along with three levels of quality control samples (low, medium, and high concentrations) included in the analysis. The performance of these quality control samples is carefully monitored using the MetVAL module in the MetIDQ^®^ software. This module compares the actual results from the quality control samples against expected results, ensuring that the measurements are consistent and accurate.

The initial metabolite dataset provided by Biocrates was subjected to a multi-stage filtering process based on the established LOD. Initially, values falling outside the LOD were treated as missing. To ensure robust analysis, metabolites were removed if more than 70% of their values were either below or above the LOD. Additionally, metabolites exhibiting constant values across all samples were excluded. For the retained metabolites, values below the LOD were imputed with the minimum detected value for that specific metabolite, and values above the LOD were replaced with the maximum detected value. This imputation method aimed to minimize bias while preserving data integrity. Each tissue type (subcutaneous fat, liver, meat, and plasma) was processed independently, yielding final metabolite counts of 168, 181, 176, and 177, respectively. Prior to mixOmics analysis, the datasets were log_2_(x + 1) transformed using R (v.4.4.0) to achieve data normalization. The final processed datasets are provided in Additional files 7–10, corresponding to subcutaneous fat, liver, meat, and plasma, respectively.

### Multi-OMICs and multi-tissue integration

It is worth noting that the data presented in this study have been previously utilized for other types of integration (Polizel, Diniz, et al. 2025; Polizel et al. 2024). However, this is the first time that all the data have been analyzed simultaneously using this specific approach. The multi-OMICs and multi-tissue integration analysis was performed using the DIABLO module (Singh et al. 2019) from mixOmics package v.6.28.0 (Rohart et al. 2017) within the R statistical environment v.4.4.0. All data, except for transcriptome data, which has higher dimensionality compared to metabolome and metagenome data, were included in the analysis after pre-processing, filtering, and normalization according to the standards of each respective -OMICs layer. For the liver and muscle transcriptomes, an additional filtering step was applied to reduce dimensionality (|log fold change| > 1.25 in at least one contrast), as described in the previous Sect. (2.4). This additional step was necessary due to the limitation of mixOmics in handling very high-dimensional data and was performed in accordance with the mixOmics guidelines. Each -OMICs × tissue combination was labeled with a specific acronym based on its tissue and -OMICs category. The labeling was as follows: liver transcriptome = Genes_LI, muscle transcriptome = Genes_MU, fecal metagenome = ASVs_FE, rumen fluid metagenome = ASVs_RU, liver metabolome = Metab_LI, meat metabolome = Metab_ME, subcutaneous fat metabolome = Metab_SF, and plasma metabolome = Metab_PL.

**Fig. 1:**
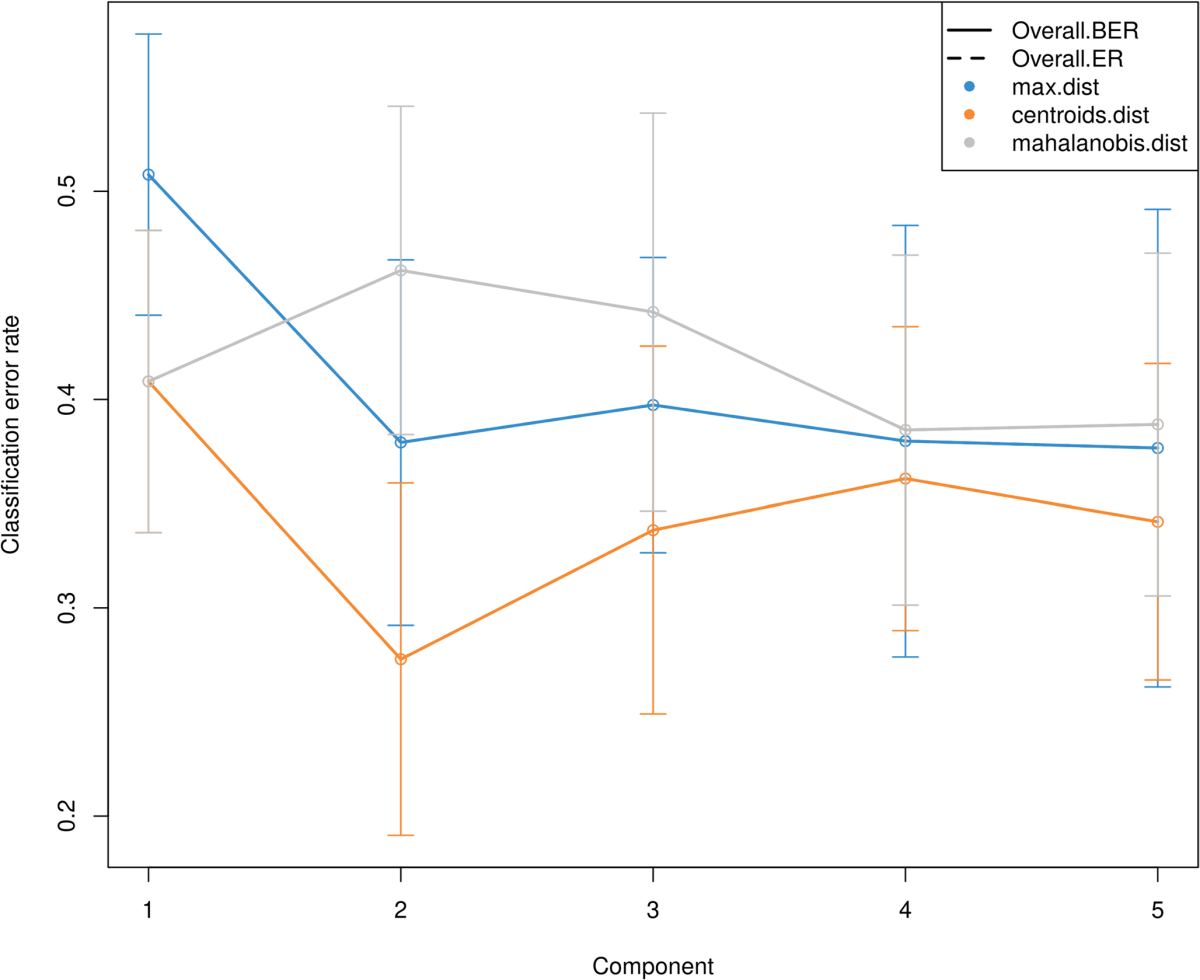
Performance plot showing the optimal number of components (X-axis) with their corresponding error rates (Y-axis), along with the best distance metric for enhancing model prediction accuracy

**Fig. 2:**
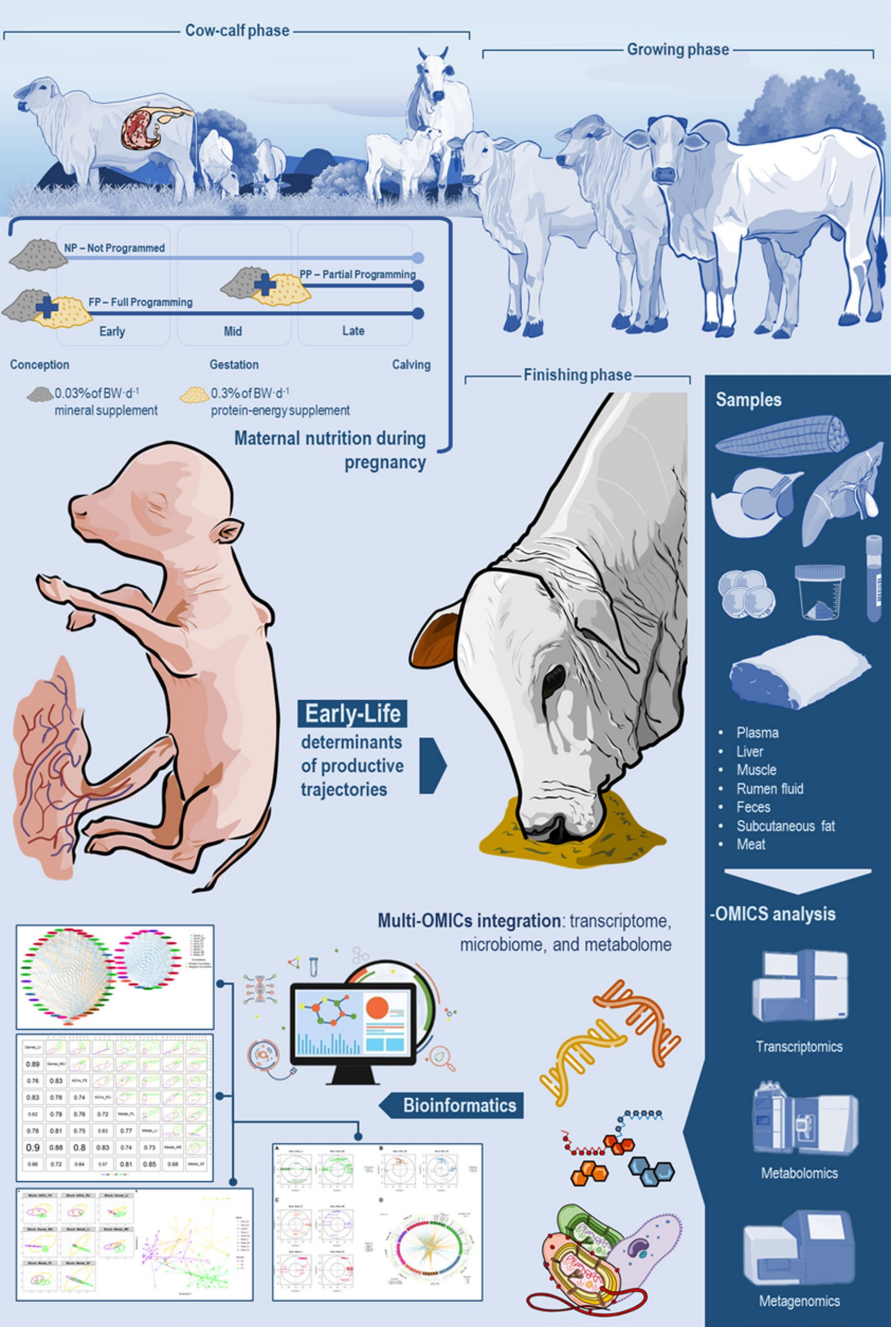
Overview of the experimental design and multi-OMICs and multi-tissue analyses

Supervised machine learning analysis was carried out using the “block.splsda” function, selecting the first two components and a 0.7 design matrix. This design matrix was computed to enhance the correlation between the components of each dataset, derived from sparse Partial Least Squares (sPLS) regression (“spls” function). The optimal number of components was determined through the “perf” function, which used centroid distance and Mfold validation. Feature selection was performed with the “tune.block.splsda” function, also utilizing Mfold validation, and tested 160,000 models to identify the potential biomarkers. Each -OMICs × tissue was considered as one block. For the correlation analysis between each block, we focused exclusively on the first component, as most blocks demonstrated a higher explained variance in this component compared to the second. However, for subsequent analyses, two components were considered. This decision was guided by the centroid distance and Mfold validation results from the “perf” function (Fig. 1). An overview of the experimental design, including nutritional treatments, sample collection, and multi-OMICs analyses, is summarized in the graphical abstract (Fig. 2).

Given the limited number of samples per group (*n* = 5), the DIABLO framework in mixOmics was used because it can handle high-dimensional and heterogeneous data blocks measured on the same samples. DIABLO extends sparse generalized canonical correlation analysis (sGCCA) to maximize both covariance between datasets and discrimination among groups. This multiblock structure allows each -OMICs layer to contribute to shared latent components, even when variable numbers differ substantially across blocks. Sparsity penalties were applied to retain the most informative features and reduce overfitting, while a design matrix was used to balance correlations between blocks. The analysis was performed in an exploratory context to identify biologically relevant associations rather than to construct a predictive model.

## Results

### Exploratory sample analysis and multi-OMICs and multi-tissue correlation via multiblock sPLS-DA for data integration

**Fig. 3:**
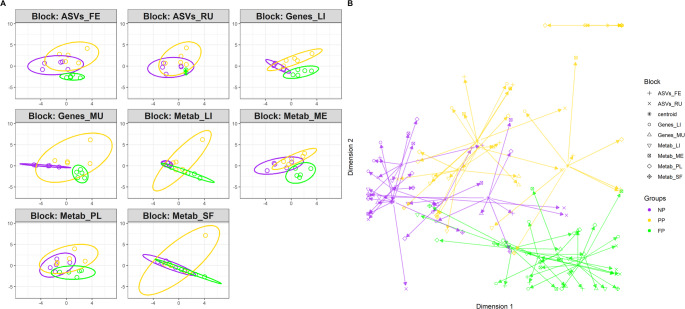
Sample plot from multiblock sPLS-DA. The samples are plotted according to their scores on the first two components for each data set. Samples are colored by maternal nutrition groups (NP, PP and FP). The plot shows the degree of agreement between the different data sets and the discriminative ability of each data set. **A** Each block represented individually. **B** Arrow plot integrating all blocks, where the base of each arrow represents the centroid across all data sets for a given sample, and the arrow tip indicates the sample’s position within each individual block

By evaluating the dimensional reduction with two latent components, we observed that the Metab_SF block accounted for the largest proportion of data variability (Component 1 = 34.04%, Component 2 = 15.18%; Table 2). However, no clear distinction was evident among the maternal nutrition groups in this block (Fig. 3A). Conversely, the metagenome blocks exhibited the lowest total explained variances (less than 20% of the total variance explained in both blocks), yet the ASVs_FE block showed a good separation between the FP group and the other groups (NP and PP). Other blocks, such as Genes_LI, Genes_MU, and Metab_ME, demonstrated clearer distinctions among the groups, and higher explained variance than metagenome blocks.

**Table 2:** Explained variance for each component and the total variance explained by both components across each -OMICs × tissue combination

Blocks	Component 1	Component 2	Total variance explained
Genes_LI	20,41%	8,00%	28,41%
Genes_MU	24,14%	9,33%	33,47%
ASVs_FE	8,66%	10,18%	18,83%
ASVs_RU	9,27%	6,38%	15,64%
Metab_PL	29,81%	5,22%	35,02%
Metab_LI	9,34%	18,91%	28,25%
Metab_ME	15,73%	23,46%	39,19%
Metab_SF	34,04%	15,18%	49,22%

Liver transcriptome = Genes_LI; Muscle transcriptome = Genes_MU; Fecal metagenome = ASVs_FE; Rumen fluid metagenome = ASVs_RU; Plasma metabolome = Metab_PL; Liver metabolome = Metab_LI; Meat metabolome = Metab_ME; and subcutaneous fat metabolome = Metab_SF

When considering the integrative approach (Fig. 3B), a global distinction between the groups becomes apparent, with the PP group displaying greater variability compared to the others. Although some individual blocks do not exhibit clear separations among the groups, integrating the data enhances the discrimination of maternal nutrition groups, thereby amplifying the overall distinction.

**Fig. 4:**
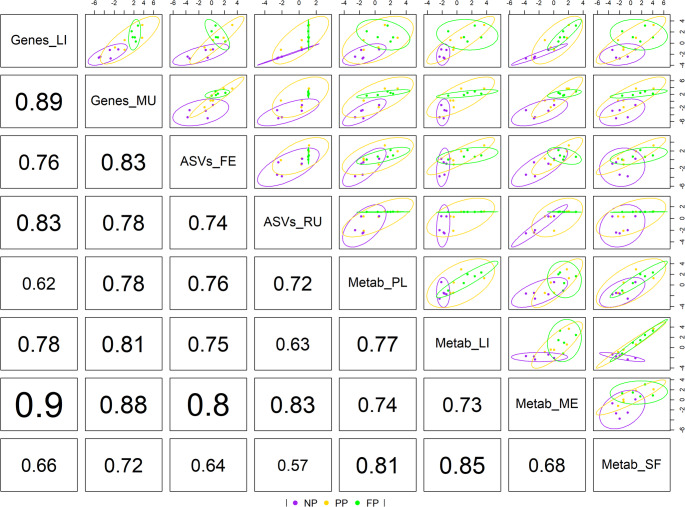
Correlation component plot from multiblock sPLS-DA. Samples are colored by maternal nutrition groups (NP, PP and FP) and 95% confidence ellipse plots are represented. The bottom-left numbers represent the Pearson correlation coefficients for the first components between each dataset

As shown in Table 2, most blocks exhibited a higher explained variance in component 1 compared to component 2. Therefore, to evaluate the correlations between the blocks, we utilized the first latent components, as illustrated in Fig. 4.

The first latent components of most blocks showed high correlations, with clear clustering observed between NP group and FP group. In contrast, the PP group exhibited overlap with other groups, indicating less distinct separation. The highest correlation was observed between Genes_LI and Metab_ME (*r* = 0.90), while the lowest was between ASVs_RU and Metab_SF (*r* = 0.57). In addition, the transcriptome blocks displayed a very high correlation with each other (*r* = 0.89), and Genes_MU showed a correlation of 0.88 with Metab_ME. Only six of the 28 pairwise correlations fell below 0.7 (*r* < 0.7), indicating a robust overall correlation structure. This analysis highlights the sPLS-DA’s ability to effectively differentiate the NP and FP groups based on the strong inter-dataset correlations.

### Multi-OMICs and multi-tissue variable correlations

In this section, rather than focusing on the sample distribution or the correlation between the entire datasets, we examined each variable within each block and analyzed the correlations of each variable across blocks. The contribution of each candidate biomarker to each component were assessed (Fig. 4A and B, and 4C,). The closer a variable is to the outer circle, the more important it is. Regions with similar variable distributions across and within blocks indicate strong correlations between those variables.

**Fig. 5:**
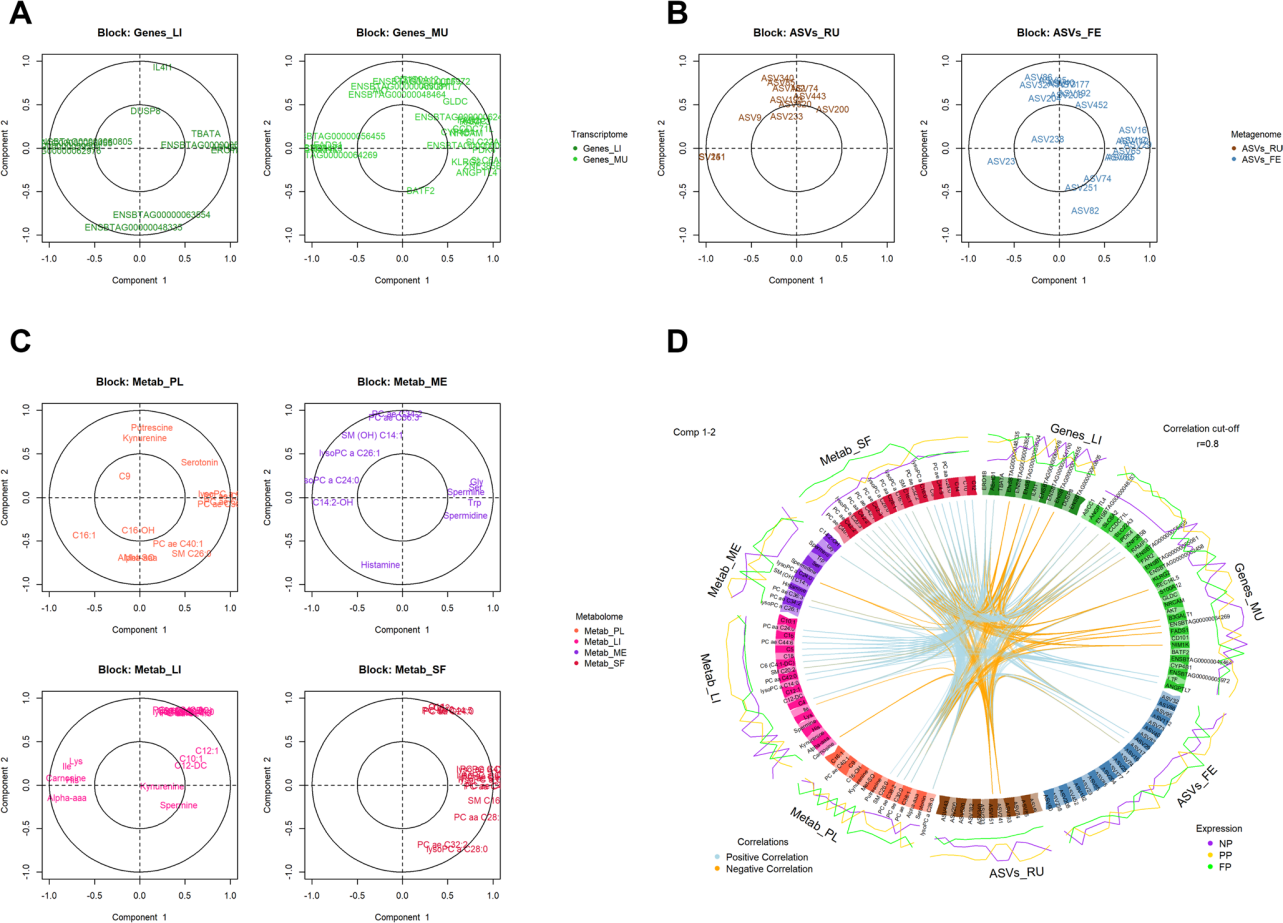
The correlation circle plot highlights the contribution of each selected variable to each component, while the circos plot shows the relationships across all -OMICs × tissues. Specifically, **A** displays the correlation circle plot of transcriptome blocks, **B** the correlation circle plot of metagenome blocks, and **C** the correlation circle plot of metabolome blocks. Lastly, **D** illustrates the circos plot, showing correlations between variables from different blocks and their expression levels across maternal nutrition groups (NP, PP, and FP)

The transcriptome blocks (Fig. 5A) showed a greater concentration of variable clusters associated with the first component negatively and positively correlated. Among them, important genes, such as *TBATA* and *ERO1B* genes in the liver and *SLC22A3* and *SLC6A2* genes in the muscle stood out. Other interesting variables, such as *IL4I1* gene (liver) and a cluster including *S100A12* and *CD101* genes (muscle), showed high importance in component 2 in liver and muscle, respectively.

The ASVs_RU block exhibited two main clusters, one of which included two key variables negatively correlated with component 1 (ASV151 = *NK4A214 group* genus and ASV241 = *Christensenellaceae R-7 group* genus). In contrast, the second cluster, associated with component 2, featured a larger number of variables linked to significant metacommunities and positive correlations, such as ASV85 (*Methanobrevibacter* genus) and ASV340 (*Aminicella* genus). For feces, although the variables showed a more dispersed distribution, two main clusters were evident. Both were positively correlated, with one cluster containing variables associated with component 1 (e.g., ASV29 = *Paeniclostridium* genus) and the other cluster containing variables associated with component 2 (e.g., ASV86 = *[Ruminococcus] gauvreauii group* genus).

Regarding the metabolome dataset (Fig. 5C), Metab_PL revealed a significant cluster in the first component, characterized by a strong positive correlation among lysophosphatidylcholine acyl C28:0 (lysoPC a C28:0), phosphatidylcholine acyl-alkyl C38:1 (PC ae C38:1), phosphatidylcholine acyl-alkyl C30:0 (PC ae C30:0), and phosphatidylcholine acyl-alkyl C38:2 (PC ae C38:2). In contrast, other variables are more dispersed across the components, exhibiting lower correlation with the second component compared to the well-defined cluster mentioned above. Metab_ME showed a positive correlated cluster with the first component (glycine [Gly], serine [Ser], Spermine, tryptophan [Trp] and Spermidine), and some positive (phosphatidylcholine acyl-alkyl C34:2 [PC ae C34:2] and phosphatidylcholine acyl-alkyl C36:3 [PC ae C36:3]) and negative (Histamine) correlated variables in component 2. Metab_LI revealed an important negative correlated cluster in component 1 composed by lysine (Lys), Isoleucine (Ile), carnosine, histidine (His) and alpha aminoadipic acid (Alpha-aaa), and another, but positive correlated with component 2 (butyrylcarnitine [C4], lysophosphatidylcholine acyl C14:0 [lysoPC a C14:0], and others). Lastly, Metab_SF exhibited two distinct clusters with strong positive correlations. The first cluster, associated with component 1, comprised variables exclusively related to glycerophospholipids. The second cluster, linked to component 2, included variables from both the acylcarnitine and glycerophospholipid classes.

To provide a broader visualization of the correlations and expression levels across maternal nutrition groups, we created Fig. 5D. Overall, while correlations above |0.8| are concentrated in a few variables within -OMICs, such as the metagenome (ASVs_FE and ASVs_RU), the metabolome and transcriptome blocks exhibited a more widespread distribution of correlations.

**Fig. 6:**
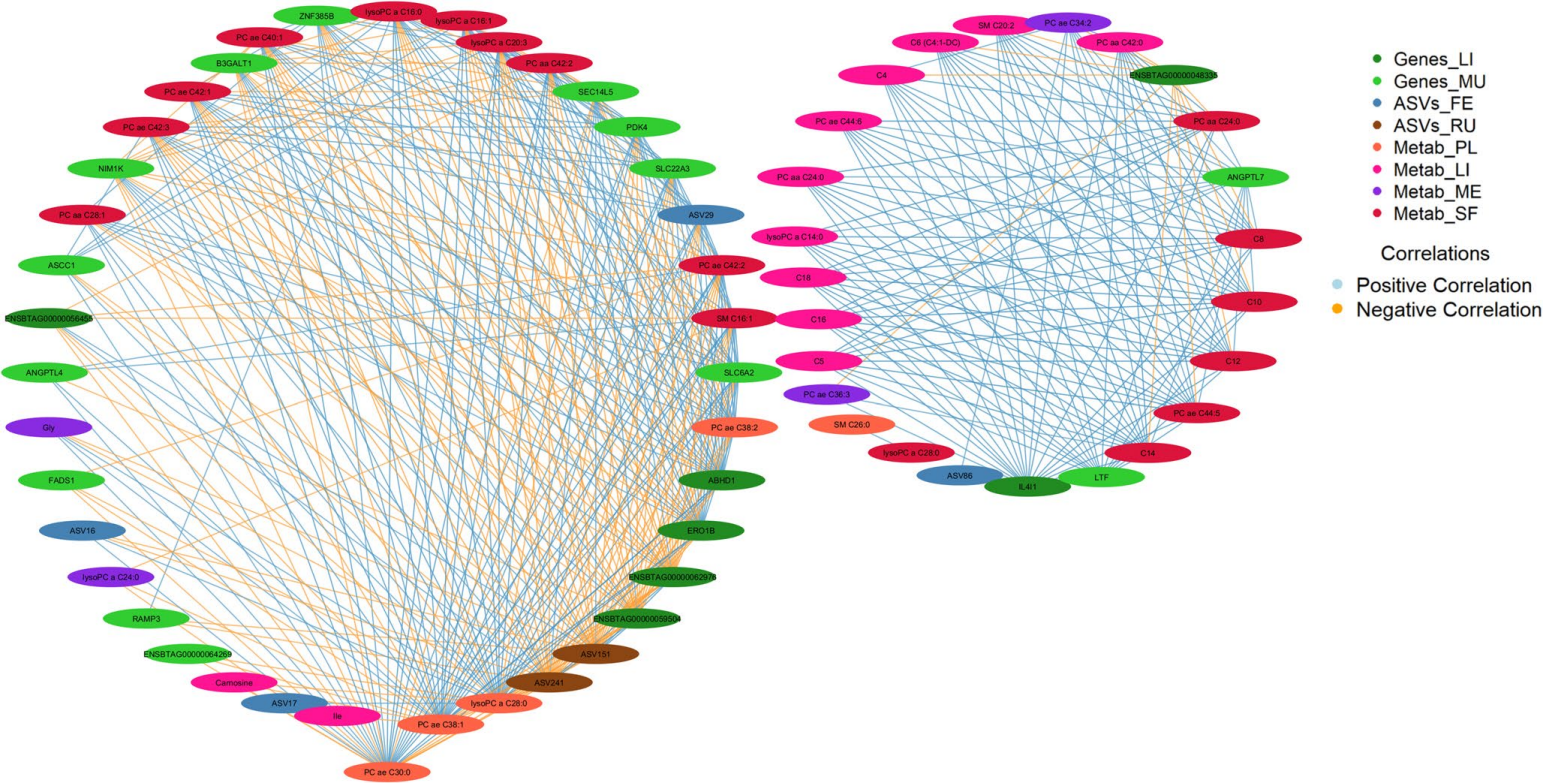
Network representation of correlations above |0.8|, visualized using a circular degree-sorted layout across the -OMICs × tissue blocks. To enhance clarity, the hub variable in the network has been slightly distanced from the others to better highlight its central role

Investigating the hub components and potential drivers of maternal nutrition effects (Fig. 6), we found that Metab_PL plays a significant role, highlighting three key variables with the highest number of connections: PC ae C30:0, PC ae C38:1, and lysoPC a C28:0. These variables exhibited connection degrees of 34, 32, and 32, respectively. Overall, Metab_ME and ASVs_FE exhibited the lowest connectivity levels among the blocks.

### Candidate biomarkers

**Fig. 7:**
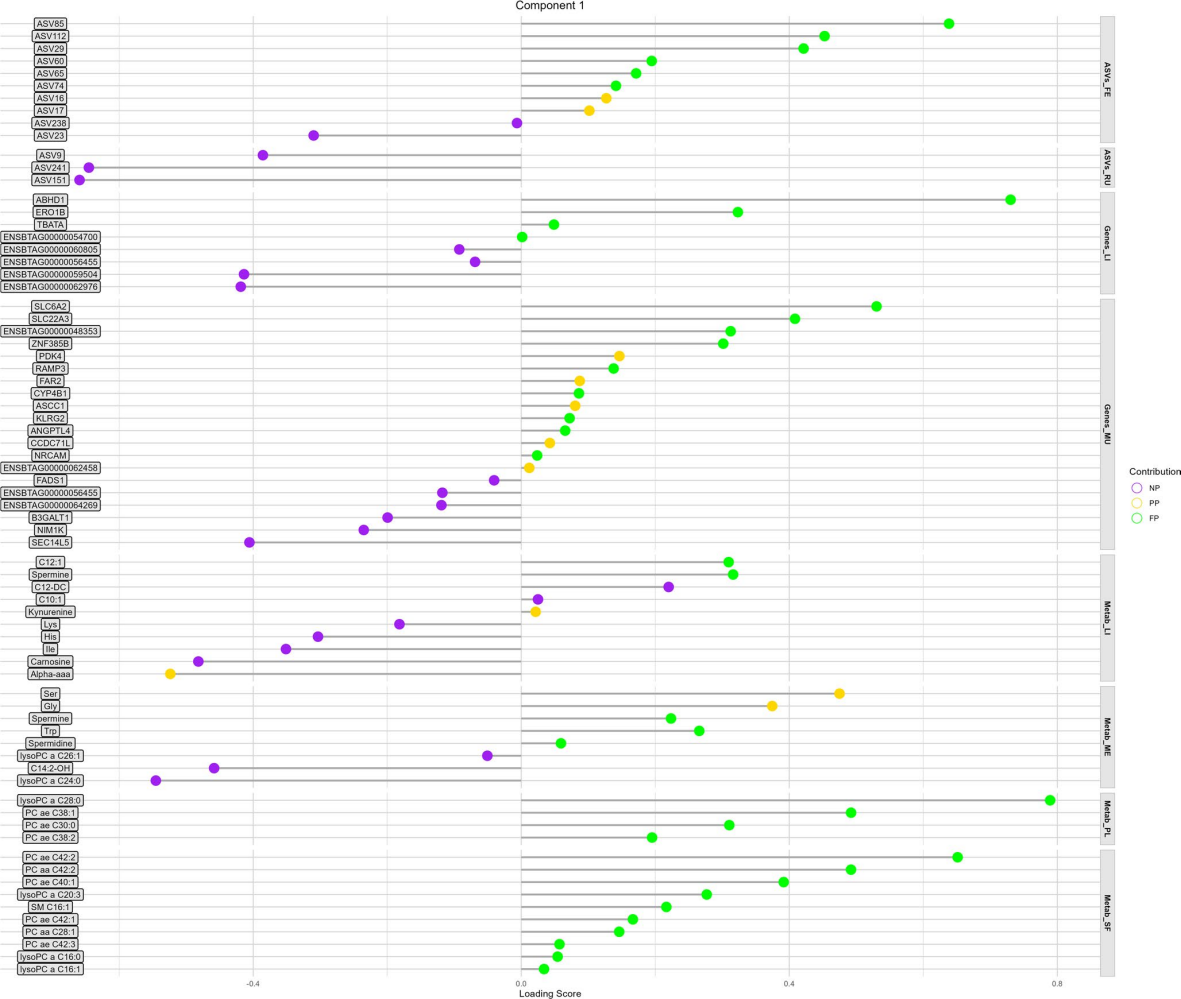
Lollipop chart representing the potential candidate biomarkers in each -OMICs-tissue block with their respective loading scores in component 1. The color of each lollipop indicates the maternal nutrition group in which the variable is a potential candidate biomarker

**Fig. 8:**
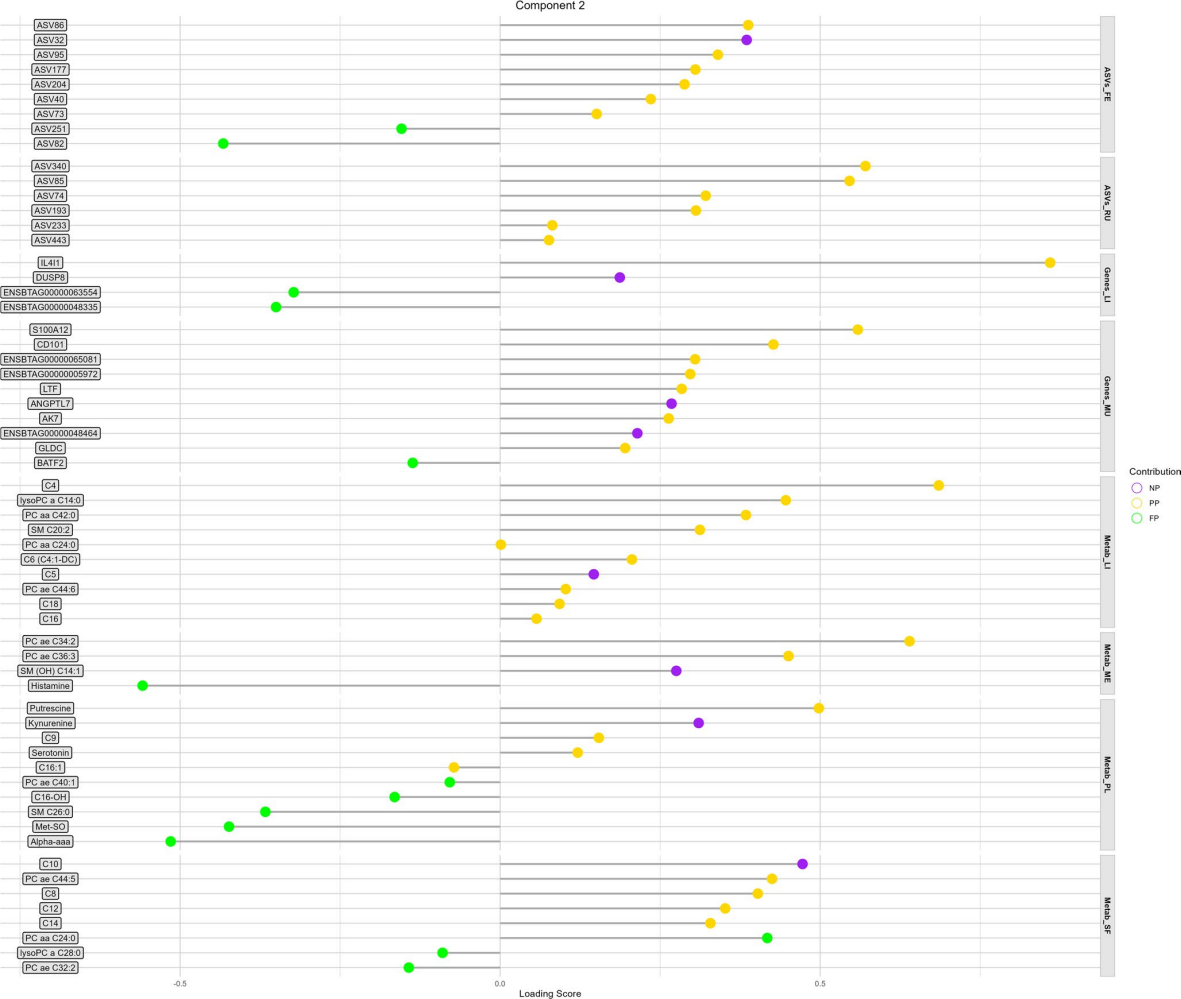
Lollipop chart representing the potential candidate biomarkers in each -OMICs-tissue block with their respective loading scores in component 2. The color of each lollipop indicates the maternal nutrition group in which the variable is a potential candidate biomarker

In general, 84.9% of the biomarkers identified in Component 1 are associated with the NP or FP group (Fig. 7), while 63.9% of the biomarkers in Component 2 are linked to the PP group (Fig. 8). Among all blocks and components, ASV151 (*NK4A214 group* genus) and ASV241 (*Christensenellaceae R-7 group* genus) exhibited the highest loading scores for distinguishing the NP group, with − 0.659 and − 0.645, respectively. The third highest loading score for the NP group was observed in the Metab_ME block, where lysophosphatidylcholine acyl C24:0 (lysoPC a C24:0) recorded a score of −0.545.

For the PP group, the liver (Genes_LI and Metab_LI) stood out, with the IL4I1 gene and C4 showing the highest scores of 0.859 and 0.685, respectively (Fig. 8). Additionally, PC ae C34:2 from Metab_ME also exhibited a high loading score of 0.639, suggesting its potential as a biomarker for the PP group (Fig. 8).

In the FP group, all variables predicted in component 1 for Metab_PL and Metab_SF were associated with the FP group. Notably, lysoPC a C28, PC ae C38:1, and PC ae C30:0 from Metab_PL emerged as candidate biomarkers, with loading scores of 0.789, 0.492, and 0.310, respectively (Fig. 7). Similarly, most biomarkers predicted in Metab_SF were linked to the glycerophospholipid class, with PC ae C42:2 displaying the highest loading score (0.651; Fig. 7).

## Discussion

Several studies have highlighted the value of using multi-OMICs approaches to investigate various phenotypes and conditions (Xiang et al., 2024; Young et al., 2023; Zhou et al., 2024). In particular, some research has employed integrative methodologies similar to those used in the present study, demonstrating their effectiveness in providing a more comprehensive and integrative understanding of biological systems (Onarman Umu et al., 2024; Ponsuksili et al., 2020; Ribeiro et al., 2023). This holistic approach not only enables the correlation of different molecular layers but also serves as a powerful strategy for predicting potential biomarkers, which are important for early diagnosis, disease monitoring, and therapeutic targeting. In this study, the identified biomarkers are considered key components linked to each prenatal nutrition strategy. These biomarkers hold significance in distinguishing between the prenatal nutrition approaches the bulls received, even 22 months postnatal. Through the visualization of sPLS-DA in each block via the sample plot (Fig. 3A) and the arrow plot when integrating the data (Fig. 3B), we observed a clear distinction in the clustering of each group. While some individual blocks do not show a clear separation of the groups (i.e., Metab_SF, Metab_LI and ASVs_RU), the integration of all blocks enhances the clustering, emphasizing the strength of a multi-block approach in revealing biological patterns that may not be apparent in single-OMICs analyses. Furthermore, as previously described, it can help identify key variables that differentiate offspring based on maternal nutrition, an insight that could be particularly beneficial for traceability in beef meat production, for example. Throughout this discussion, we have mainly focused on the potential biomarkers with the highest scores and their role within a systems biology context, as well as the correlations between different -OMICs × tissue.

The muscle transcriptome (Genes_MU) emerged as the only dataset exhibiting exclusively strong correlations (0.72 ≤ |r| ≤ 0.89) [36], reinforcing its potential as the most representative -OMICs × tissue combination in this study. This finding may be attributed to the lower priority of muscle in nutrient partitioning during pregnancy (Du et al., 2010; Sookoian et al., 2013), potentially rendering it more predisposed to the influences of prenatal nutrition compared to tissues like the liver or brain. Similarly, the meat metabolome (Metab_ME) displayed strong correlations but also showed a very strong correlation with Genes_LI (*r* = 0.90) and one correlation below the strong threshold with Metab_SF (*r* = 0.68). Overall, all blocks exhibited at least moderate correlations (0.40 ≤ *r* ≤ 0.69), underscoring the representative power of Genes_MU within a systems biology context and its effectiveness in distinguishing between the NP and FP maternal nutrition groups across all comparisons based on the first latent components.

Our results identified PC ae C30:0, PC ae C38:1, and lysoPC a C28:0 from plasma (Metab_PL) as hub metabolites, each displaying more than 30 correlations above |0.8| across all blocks. This strong connectivity highlights their central role in the metabolic network of long-term effect of maternal nutrition. A multi-OMICs study investigating the impact of seasonal weight loss tolerance on goat mammary gland metabolism reported similar findings, identifying metabolites as key driving molecules (Ribeiro et al., 2023). Unlike their study, which focused on only three blocks (transcriptome, proteome, and metabolome), our study incorporates four distinct metabolome tissues, enabling us to infer which specific metabolome tissue is most suited to act as a central regulator. Blood plays a key role in linking biological systems at both the physiological and metabolic levels due to its continuous interaction with the entire system (Liew et al., 2006). This makes it expected that the top three molecules with the highest number of connections originate from plasma tissue. Interestingly, all three plasma metabolites, acting as metabolic drivers, are potential candidate biomarkers for the FP group, with lysoPC a C28:0 showing the highest predictive score. In the first component, Metab_PL and Metab_SF were identified as the most suitable tissues for identifying potential biomarkers in the FP group, with all associated molecules belonging to glycerophospholipids and one to sphingolipids.

The glycerophospholipids are essential components of cell membranes and play important roles in cell signaling, membrane fluidity, and metabolic regulation (Hishikawa et al. 2014; Im 2013; Mukhopadhyay et al. 2022). Their involvement in several biological pathways suggests a potential link between lipid metabolism in both plasma and subcutaneous fat and the FP prenatal treatment group. This reinforces the idea that alterations in lipid metabolism in these tissues may be a defining feature of the FP group, further supporting the potential of these metabolites as biomarkers for its identification. Importantly, in our previous study, we identified the effects of maternal nutrition on lipid metabolism and observed an association between the FP group and the higher level of glycerophospholipids (Fernandes, Henrique, et al. 2023; Polizel, Diniz, et al. 2025), highlighting the lasting impact of early-life nutritional influences on metabolic regulation in beef cattle. The higher abundance of glycerophospholipids observed in both subcutaneous fat and plasma of the FP group may reflect reduced catabolism for skeletal muscle energy expenditure, instead promoting their retention and incorporation into adipocyte membranes. Since glycerophospholipids serve as fundamental structural components for cellular membranes, their accumulation in adipose tissue likely supports enhanced adipocyte membrane biogenesis during subcutaneous fat development.

Regarding the potential biomarkers for protein-energy supplementation during the last third of gestation (PP group), we found that the two variables with the highest loading scores were associated with liver tissue (Genes_LI and Metab_LI). Among these, the interleukin-4 induced gene 1 (*IL4I1*) stood out, showing the highest loading score across all blocks and treatments. Additionally, *IL4I1* was the most interconnected variable in the secondary network generated (Fig. 6), further reinforcing its potential as a strong candidate biomarker for the PP group. The *IL4I1* encodes an enzyme with L-amino acid oxidase activity, primarily catalyzing the oxidative deamination of L-phenylalanine, and to a lesser extent, L-arginine, under physiologically optimal pH conditions (Boulland et al., 2007). This enzymatic activity plays a key role in modulating immune responses and amino acid metabolism (Romagnani, 2016). The gene’s high degree of connectivity and consistent relevance across multiple data layers suggest it may be a central player in the metabolic and immunological adaptations induced by late-gestation supplementation. Moreover, a previous integrative study of the liver transcriptome and metabolome revealed significant associations between the PP group, arginine metabolism, and immune function (Polizel et al., 2024). These findings highlight a potential mechanistic link between the late gestation maternal nutrition and immune-metabolic regulation. Further investigation into *IL4I1*’s functional role could provide deeper insights into these processes and reinforce its potential as a biomarker of PP group.

Butyrylcarnitine (C4) also emerged as a potential biomarker to the PP group. Acylcarnitines are traditionally analyzed in neonatal screening to detect inborn errors of metabolism (López-Suárez et al., 2019). However, disruptions in acylcarnitine profiles are not limited to inherited metabolic disorders; they may also reflect a variety of pathological conditions, including sepsis, malnutrition, type I and II diabetes, obesity, and intrauterine growth restriction (Bene et al., 2013; Fernandez-Lainez et al., 2012; Liu et al., 2016; Mihalik et al., 2010; Neugebauer et al., 2016). This diagnostic versatility is attributed to the biochemical properties of L-carnitine, a semi-essential nutrient for newborns (Crill et al., 2006; Pande et al., 2005), whose free hydroxyl group can esterify with a broad spectrum of acyl groups. This allows acylcarnitines to serve as intermediates in the transport and metabolism of fatty acids and other metabolites (Sánchez-Pintos et al., 2016). In the context of increased protein-energy supplementation exclusively during the final third of gestation, the metabolic shift from a preceding period of low protein-energy intake to a sudden nutritional increase may favor the accumulation or modulation of C4. This shift may also highlight the liver’s central role in coordinating the metabolic adaptations observed in the PP group. We propose this as a possible explanation for the emergence of C4 as a potential biomarker, reflecting the liver’s adaptive response to late gestational nutritional intervention.

In the first component, the rumen fluid metagenome (ASVs_RU) stood out, with all candidate biomarkers identified in this tissue being associated with the NP group. Notably, two of these biomarkers exhibited both the highest loading scores and the greatest linkage degrees within the network, highlighting their central role. These findings suggest that, following plasma, the rumen fluid metagenome is a key driver in the response to prenatal nutrition, particularly in distinguishing the NP group. The ASV151 (*NK4A214 group* genus) and ASV241 (*Christensenellaceae R-7 group* genus) are both classified within the class *Clostridia* and have been associated with the production of the short-chain fatty acids propionate and butyrate (Low et al., 2022; Sun et al., 2019). In ruminants, volatile fatty acids contribute up to 70% of the total energy supply (Baldwin & Connor, 2017). Unlike acetate fermentation, which generates hydrogen as a byproduct, the formation of propionate and butyrate in the rumen involves hydrogen consumption, a process considered to be more energy-efficient (Auffret et al., 2020; Kruger Ben Shabat et al., 2016). The negative loading scores associated with these metacommunities suggest that animals in the NP group may exhibit lower energy efficiency. This interpretation is further supported by our previous findings (Dias et al., 2024), which showed that the NP group had the lowest number of ruminal papillae, indicating reduced absorptive capacity and, consequently, less efficient energy utilization.

It is worth noting that although the integrated analysis was based on a limited number of animals, the DIABLO framework in mixOmics is designed to handle heterogeneous and high-dimensional data using sparse modeling and cross-validation to limit overfitting. Thus, our results should be viewed as exploratory and hypothesis-generating, yet the consistent multi-OMICs patterns observed across tissues suggest biologically meaningful associations that warrant further validation in larger cohorts.

## Conclusions

The integrative multi-OMICs and multi-tissue approach provided a more comprehensive understanding of the biological responses to maternal nutrition strategies compared to single-layer analyses. This integrative framework not only improved the discrimination between nutritional groups but also revealed key tissue- and OMIC-specific patterns. The muscle transcriptome (Genes_MU) demonstrated consistent cross-block correlations, reinforcing its role as a representative tissue to maternal nutritional modulation in beef cattle. The identification of plasma glycerophospholipids (Metab_PL) among the most connected variables across all blocks reinforces their role as potential biomarkers for the FP group and highlights a possible link between full prenatal supplementation and lipid metabolism. In the PP group, the liver-associated biomarkers *IL4I1* and C4 point to a coordinated immune-metabolic adaptation driven by late gestation supplementation. Meanwhile, the NP group was characterized by a reduced abundance of ruminal *Clostridia* (ASV151 and ASV241), suggesting a potential compromise in microbial-driven energy efficiency. Collectively, these findings underscore the value of integrative multi-OMICs analyses in identifying candidate biomarkers associated with distinct maternal nutrition in beef cattle.

## Supplementary Information

Below is the link to the electronic supplementary material.

All the Additional files (1 to 10) are presented in Supplementary material 1. Supplementary material 1 (XLSX 292.1 kb)

## Data Availability

The transcriptome datasets used in this study are available in the supplementary material (Additional file 1: liver; Additional file 2: muscle). The metagenomic datasets are provided in Additional file 3 (fecal metagenome) and Additional file 4 (rumen fluid metagenome), along with their respective taxonomic classifications in Additional file 5 (fecal taxonomy) and Additional file 6 (rumen fluid taxonomy). All metabolomic datasets are also included as supplementary material: Additional file 7 (subcutaneous fat), Additional file 8 (liver), Additional file 9 (meat), and Additional file 10 (plasma). All supplementary data have been compiled into a single Excel file, organized into 10 separate sheets corresponding to each dataset.Additionally, the raw transcriptome data have been deposited in the European Nucleotide Archive (ENA) repository (EMBL-EBI), under accession numbers PRJEB84398 (muscle) [http://www.ebi.ac.uk/ena/browser/view/PRJEB84398] and PRJEB75582 (liver) [http://www.ebi.ac.uk/ena/browser/view/PRJEB75582].

## Abbreviations

Alpha-aaa: Alpha aminoadipic acid
ASV151: NK4A214 group genus
ASV241: Christensenellaceae R-7 group genus
ASV29: Paeniclostridium genus
ASV340: Aminicella genus
ASV85: Methanobrevibacter genus
ASV86: [Ruminococcus] gauvreauii group genus
ASVs: Amplicon sequence variants
ASVs_FE: Fecal metagenome
ASVs_RU: Rumen fluid metagenome
BW: Body weight
C4: Butyrylcarnitine
CP: Crude protein
DIABLO: Data integration analysis for biomarker discovery using latent components
FIA-MS/MS: Flow injection analysis-tandem mass spectrometry
FP: Full programming
Genes_LI: Liver transcriptome
Genes_MU: Muscle transcriptome
Gly: Glycine
His: Histidine
HPLC: High-performance liquid chromatography
HPLC-MS/MS: Liquid chromatography tandem-mass spectrometry
IL4I1: Interleukin-4 induced gene 1
Ile: Isoleucine
LOD: Limits of detection
Lys: Lysine
lysoPC a C14:0: Lysophosphatidylcholine acyl C14:0
lysoPC a C24:0: Lysophosphatidylcholine acyl C24:0
lysoPC a C28: Lysophosphatidylcholine acyl C28:0
lysoPC a C28:0: Lysophosphatidylcholine acyl C28:0
MAPA: Ministry of agriculture, livestock, and supply of Brazil
Metab_LI: Liver metabolome
Metab_ME: Meat metabolome
Metab_PL: Plasma metabolome
Metab_SF: Subcutaneous fat metabolome
NP: Not programmed
PC ae C30:0: Phosphatidylcholine acyl-alkyl C30:0
PC ae C34:2: Phosphatidylcholine acyl-alkyl C34:2
PC ae C36:3: Phosphatidylcholine acyl-alkyl C36:3
PC ae C38:1: Phosphatidylcholine acyl-alkyl C38:1
PC ae C38:2: Phosphatidylcholine acyl-alkyl C38:2
PC ae C42:2: Phosphatidylcholine acyl-alkyl C42:2
PP: Partial programming
r: Correlation
RIN: RNA integrity number
Ser: Serine
sPLS: Sparse partial least square
TDN: Total digestible nutrients
Trp: Tryptophan
